# Willingness to use couples HIV testing and discussion of sexual agreements among heterosexuals

**DOI:** 10.1186/s40064-015-0939-1

**Published:** 2015-04-08

**Authors:** Rob Stephenson, Catherine Finneran, Tamar Goldenberg, Patricia Coury-Doniger, Theresa E Senn, Marguerite Urban, Ann Schwartz, Patrick Sullivan

**Affiliations:** Department of Health Behavior and Biological Sciences, School of Nursing, University of Michigan, Ann Arbor, MI 48109 USA; Department of Epidemiology, Rollins School of Public Health, Emory University, Atlanta, GA 30322 USA; Center for Health and Behavioral Training, Infectious Diseases Division, University of Rochester Medical Center, Rochester, NY USA

**Keywords:** HIV, Heterosexual, HIV testing, Couples, Sexual agreements

## Abstract

Couples HIV Testing and Counseling (CHTC) has been used as an HIV prevention strategy in Africa for over 20 years where the HIV epidemic is largely concentrated among sexually active heterosexuals. In recent years, CHTC has been adapted for men who have sex with men (MSM) in the US. A central element of the CHTC intervention as adapted for male couples in the US is the discussion of sexual agreements by the dyad during the CHTC session. Given the success of CHTC for heterosexual couples in Africa, it seems appropriate that CHTC could also be provided to heterosexual couples in the US. However, little is known about heterosexual’s willingness to utilize CHTC services including discussion of sexual agreements. This small, preliminary qualitative study sheds new light on the potential for CHTC adoption among heterosexuals in the US. Four focus groups were conducted with heterosexual men and women attending a publicly-funded STI clinic, to explore the potential feasibility and acceptability of CHTC with heterosexuals. The results are similar to those seen for MSM: high levels of willingness to use CHTC, perceptions of the advantages of using CHTC, and willingness to discuss sexual agreements; all necessary conditions for the successful roll-out of CHTC. Further work is now needed with larger samples of high-risk heterosexuals to more completely understand the typologies of sexual agreements and the common language used for sexual agreements in heterosexual relationships. These early data show great promise that CHTC can achieve the same levels of willingness, fit, and acceptability among heterosexual couples as currently experienced by male couples in the US.

## Introduction

Couples HIV Testing and Counseling (CHTC) has been used as an HIV prevention strategy in Africa for over 20 years where the HIV epidemic is largely concentrated among sexually active heterosexuals (Allen et al. 1992, 2003; Painter 2001). CHTC builds upon the conventional model of individual testing by allowing couples to receive all stages of the HIV testing and counseling process together: couples receive individualized HIV counseling and prevention messaging based on the characteristics of their relationships and their joint HIV status. In individual HIV counseling and testing an individual is asked retrospective questions about their risk sexual and non-sexual HIV risk exposures (e.g. number of sexual partners, condom use). In CHTC couples are not asked these retrospective questions. Rather the focus of CHTC is on the couple learning their sero-status together and making a prevention plan together with a focus on future behaviors and actions. CHTC has demonstrated success in reducing sexual risk behavior and promoting mutual disclosure of HIV status among African heterosexual sero-discordant couples (i.e., couples in which one is HIV negative and one is HIV positive) (Allen et al. 1992, 2003). In a non-randomized prospective study of heterosexual couples, HIV-negative women whose partners had not participated in CHTC had a small reduction in HIV incidence, from 4.1/100 person-years (PY) to 3.4/100 PY; the incidence rate among women whose partners had participated in CHTC was about half as high (1.8/100 PY, p < 0.04) (Allen et al. 1992). Previous studies have also demonstrated CHTC to be effective in increasing and sustaining condom use and reducing sexual risk-taking among sero-discordant heterosexual couples (Allen et al. 1992, 2003; Painter 2001; Roth et al. 2001). CHTC has received significant support through The President’s Emergency Plan for AIDS Relief (PEPFAR), has been adopted widely in sub-Saharan African countries with high adult HIV prevalence, and has been recommended by the World Health Organization (WHO) as part of an integrated testing and biomedical prevention strategy (World Health Organization 2012).

Significant research and programmatic attention has been paid recently to the adaptation and roll out of CHTC for same-sex male couples in the US. Guided by a new understanding of the epidemiology of HIV among men who have sex with men (MSM) in the US, which showed that main sex partners account for between one-third (Goodreau et al. 2012) and two-thirds of new infections (Sullivan et al. 2009) and a high observed prevalence of undiagnosed HIV infection among US MSM (CDC 2006), Sullivan et al. (2014) sought to develop a new HIV testing strategy for male couples in the United States by adapting the CHTC model originally developed for heterosexual couples in Sub-Saharan Africa. Guided by the ADAPT-ITT (Wingood and DiClemente 2008) framework (a model for adapting evidence based HIC interventions), Sullivan et al. (2008) applied a systematic, data-driven process to adapt and test CHTC for same-sex male couples. The adaptation process and results are described elsewhere (Sullivan et al. 2014); briefly, the outcome of this process was an adapted CHTC service that proved to be acceptable to MSM and HIV prevention counselors, and which did not show evidence of any harm (e.g., intimate partner violence [IPV] incidence, relationship dissolution). The adapted service has been delivered to the public health community for dissemination and scale-up by CDC, is listed on CDC’s Effective Interventions website, and is now available in over 30 US cities.

The primary difference between the African heterosexual model of CHTC and the newly adapted version for US male couples was the addition of a module on sexual agreements within the relationship. In the context of understanding HIV risk among same-sex male couples, much attention has been paid recently to the role that sexual agreements have in shaping HIV risk (Gass et al. 2012; Gomez et al. 2012; Hoff et al. 2012; Mitchell 2013a, b). Sexual agreements refer to the explicit rules established by couples governing the allowance of sex with partners outside of the relationship, and the conditions under which sex is permissible. Sexual agreements may include monogamy (sex exclusively between main partners) and open agreements (that permit sex with partners outside of the relationship) that may have conditions applied (e.g., only specific types of sex are allowed with outside partners) (Hoff and Beougher 2008; Hoff et al. 2012). Sexual agreements are common among same-sex male couples: among a sample of 732 MSM in main partnerships, 91% of respondents reported having a sexual agreement with their main partner (Gass et al. 2012). Agreements are dynamic: over time, couples may decide to change their agreement as the relationship evolves and sexual agreements may be established or renegotiated at the beginning of the relationship, at any point in the relationship when one or both partners wishes to open or close the agreement, or immediately after an agreement breakage has been disclosed (Mitchell 2013a; Hoff and Beougher 2008). The establishment of an agreement can provide structure to help define the relationship and offer a sense of security for both partners in which both feel that their sexual and emotional needs are being heard (Mitchell 2013a; Hoff and Beougher 2008). As provided to same-sex male couples in the US, CHTC now asks couples to discuss their sexual agreement and revisit the terms of their agreement in light of their HIV test results. For those who do not have an existing agreement, CHTC counselors help couples discuss the boundaries of an agreement, informed by their HIV tests results.

As part of the roll-out of CHTC in the US, the CDC Division of HIV/ AIDS Prevention (DHAP) is encouraging CHTC to be available to couples of all genders. Women make up 23% of all new HIV infections in the US. Fifty-seven percent of new HIV infections among women occur in Black women and 16% among Hispanic women (CDC 2012, 2014). The majority of HIV+ women (84%) were infected through heterosexual contact (CDC 2012, 2014); therefore, if CHTC can be adapted for heterosexual couples in the US, it may be an important tool for reducing HIV, particularly among women.

Nationally, 11% of women and 12% of men report having concurrent sexual partnerships, or partnerships that overlap in time (Adimora et al. 2002, 2007). Among urban and high-risk populations, rates of concurrency are much higher, and frequently involve a longer-term sexual relationship with a steady or primary partner, and additional non-primary or casual partners. For example, among urban STI clinic patients with a primary sexual partnership of 3 months or more, 64% reported also having a non-primary sexual partner in the past 3 months (Senn et al. 2009). Among women at high risk for HIV who reported having a primary sexual partner for at least 6 months, 42% reported having at least one additional sexual partner in the past 90 days (Grieb et al. 2012). Unlike for MSM, for whom explicit sexual agreements within primary partnerships are common and generally well-accepted, relatively little is known about whether heterosexual relationships in the US are governed by explicit or implicit sexual agreements, and whether an open discussion of sexual relationships and the establishment of an agreement about sexual relationships, which is a critical part of CHTC in the US, would interfere with the feasibility and acceptability of CHTC among heterosexuals in the US. In this paper we present preliminary qualitative data from gender stratified focus groups with heterosexual men and women, recruited through a publicly-funded STI clinic in Rochester, NY. The data are intended to inform the continued adaptation of CHTC to match the needs of the US HIV epidemic.

## Methods

Focus group discussions were used to examine male and female participants’ perceptions of CHTC, their knowledge and experience of sexual agreements, and their comfort in discussing sexual agreements in CHTC sessions. In total four focus group discussions were held: two with male participants and two with female participants. We conducted focus groups separately by gender to increase participant comfort and to encourage open and honest discussion. All four groups were facilitated by a trained moderator from Emory University. Participants were recruited through two public health clinics in Rochester, NY. The target population for the groups was men and women aged over 18 years who reported a heterosexual identity and reported sex with a member of the opposite sex in the previous six months. The aim was to have approximately 10 participants per group: anticipating subject loss, we over-recruited by approximately 25%, and offered a small monetary incentive to encourage participation. Flyers advertising the study and detailing inclusion criteria, compensation and the aims of the study were posted in each of the clinics. Potential participants who contacted the study organizers were screened on the aforementioned criteria. Upon arrival at the focus group venue, participants went through the consent process, and then completed a screening questionnaire (including age, race, and relationship status). All focus groups were audio recorded and transcribed verbatim. The development of the semi-structured question guide for the focus groups was informed by the existing literature on CHTC and sexual agreements. In total 37 participants were included in the focus groups. Of the 37 participants in the focus groups, 33 self-identified as African American and four as Caucasian. All participants were aged over 18 and ranged in age from 24–43. The majority (70%) reported being in a relationship; data on relationship length was not collected. The study was approved by the IRBs of the participating institutions.

Thematic analysis was conducted on the pooled data from the focus group transcripts, which involved the coding and classification of the data by reviewing the transcriptions for potential conceptual categories, using the focus group guide questions as initial categories (Stemler 2001). Thematic analysis entailed the consistent application of a set of codes to all verbatim transcripts in order to examine how themes were discussed between groups of participants. A preliminary codebook was created based on a close reading of several transcripts, incorporating explicit domains from interview guides (deductive themes) as well as pervasive, unanticipated themes that were emergent across various transcripts (inductive themes). Provisional definitions were given to each code and two analysts applied the codes to a single transcript. The coded transcripts were merged for comparison and code definitions were revised based on an examination of coding disagreement. This process was repeated until consistent agreement was attained among all coders. This process was conducted using MAXQDA software for qualitative data management and analysis. Comparisons were made within the data to detect divergent views by gender. Key quotes, including common and outstanding quotes statements, were chosen to represent the data.

## Results

### Relationship agreements

The discussions began with an open-ended question about relationship agreements which were described by the moderator as “*ground rules or laws for a relationship about whether or not the people in the relationship can have sex with other people*.” Although most participants reported awareness of sexual agreements, they also acknowledged that they did not generally have explicit sexual agreements with partners that are expressed verbally. Most participants reported that sexual agreements largely took the form of tacit, unspoken “*understandings*”:

“…*if it’s a relationship, I guess you got to discuss what’s the terms before you even ground what it is, but most never discuss it*” (male).

“*But like in every relationship it should be an understanding*” (male).

Implicit agreements were characterized by unspoken expectations between partners. A female participant said, “*I never went and sat down with any of my boyfriends and be like, ‘Listen these are ground rules.’ It just happened*, *because it was kind of just expected that once you consider yourself devoted to another person that that’s just that”* (female).

Central to the discussion of sexual agreements in the focus groups was the recurring theme that sexual agreements were not discussed between male–female partners: they were understood to be in place and were expected. In the second male focus group there was a distinction made between rules and “*understandings*.” Rules were expressed as based in explicit communication while “*understandings”* were reported to develop over time, often without discussion, as the relationship developed: *“But look. That ain’t a rule. That’s an understanding. You and she got an agreement, because a lot of things go without saying”* (male). Participants often reported that monogamy was not a decision to be made, but was an unspoken expectation: “*Monogamy is just expected that once you consider yourself devoted to another person that that’s just that once I pick somebody, I’m exclusive to that person*” (female). Many participants reported that they had never discussed relationship rules because they didn’t require discussion: *“it just happened*” (female). Some even suggested that setting rules somehow takes away from the quality of the relationship. One male participant said: *“When you make a rule like that’s not established relationship so it shouldn’t be in a relationship, it should just be we’re gonna do this and we’re still gonna do us. Like not, it’s not a partnership anymore once you create rules”* (male).

When probed to discuss types of sexual agreements that may occur in heterosexual relationships, participants focused exclusively on “*open relationships*” and “*threesome*s.” Open relationships (referred to by some participants as “*swinger*s”) were described generally as both partners within a dyad having an explicit agreement to be able to have sex with other people. “*Swingers*” were defined as having sex *“with other married couples or other unmarried couples”* (male). Threesomes generally were spoken about as one male and two females, and female participants overwhelming reported that threesomes (or the idea of threesomes) were instigated by male partners. Some women reported that men were usually reluctant to take part in a two males-one female threesome, which could be used as reasoning not to partake in any threesomes: “*And you know why you can kind of kick back on that, do one on one psychology, OK so you want a threesome, OK, but let me get a threesome, I want one too… I want a man, let me have one too” (*female*).* Some women reported that threesomes with two women were a way of fulfilling their male partner’s desires that they were unwilling to fulfill: “*I didn’t want to do such and such with him, or whatever, so I would go get other girls to do these acts for him. But when I went and got the other girls I would specify to them what to do and what not to do, and how far to go and how far not to go*” (female). Another female participant described a situation where she was approached to have sex with a man in a dyad whose wife wanted to watch them have sex*: “Him and his baby momma, she wasn’t satisfying him sexually, so she wanted to watch him have sex with another female to see what she’s not doing for him”* (female).

Although threesomes were discussed experientially by participants, explicit open relationships were generally described as an *idea* – that is, no participants reported experiences of open agreements, or participants cited media examples of celebrities in open relationships. Heterosexual relationships were consistently referred to as monogamous, and having outside partners usually came up in discussion in the context of infidelity: “*You really don’t sit down and say you better not be out there cheating on me because you expect him not to go out there and cheat. So you never sit down and talk*” (female). Two prominent themes around infidelity emerged. First, discussion of sexual agreements was often seen as a sign of infidelity, a theme that was more commonly reported by women. Women reported apprehension in bringing up the topic of sexual agreements in case it was viewed by their male partner as evidence of infidelity: “*He might flip it on me. I mean, like I’m out cheating, you know.*” (female). One male participant expressed disappointment from not being received well when sharing his feelings about sexual agreements: “*That effort and that, because, and even then you could turn around and get stung because you never know what a person’s capable of, no matter what kind of communication, conversation you have. And so, like he said, it’s a gamble, it’s all a roll of the dice*” (male). Both men and women mentioned third parties as helpful in mediating discussions around sexual agreements, with one male participant saying that female family members had mediated for him, and one female participant reporting: “*It might be easier for me to say something to him about our relationship with somebody else in the room*” (male). Participants also discussed how asking to use condoms after not previously using condoms or after getting an HIV test could be cause for suspicion of infidelity.

Secondly, infidelity was reported to be a common trigger for the discussion of explicit sexual agreements: “*It’s gonna come up. It might not be now but when y’all do argue about something that’s completely unrelated… you talk about I was cheating last year*” (male). When probed to discuss situations in which men or women may have ongoing concurrent sex partners in addition to their main partner, the majority of participants referenced situations in which men were having sex with other men, often talked about as *“messin.”* This was reported as exclusively male: discussions did not mention women having sex with other women. The second most common depiction of concurrency was of men having sex with other women outside of the relationship: “*My mom said, you know, she used to feel like as long as he don’t bring nothing home”* (female). Only twice was there mention of women having male sex partners outside of the relationship: “S*he might have had sex with somebody else before she had sex with you but you thinking this your broad now because she done gave you a little piece of leg, right…. She probably just out there searching … trying to find that right one but she still popping her leg”* (male).

Despite the majority of participants reporting that heterosexual relationships had “*understandings”* and not agreements, and the problems linked to infidelity noted in the discussion of sexual agreements, the majority of both male and female participants reported being in favor of having explicit sexual agreements. As one female participant noted, “*It does make it easier at the beginning and you might as well get your cards all out on the table because, like you said, you find out four, five, six, years later this is what his idea of cheating is and this is what my idea of cheating is and we’re not even on the same page…So like might as well get it out of the way*” (female). However, there were some gender differences in how men and women spoke about the content of sexual agreements. Men focused on the sexual aspects of sexual agreements, while women expanded the discussions to a broader definition of relationship agreements, including, for example, finances: “*you need money to survive, and I know what I’m not going to be doing this bill and you ain’t doing no bills, this bill in half, and it’s coming every month, yeah he’s going to pay that in full*” (female).

### Acceptability of couples voluntary counseling and testing (CHTC)

Overall, both male and female participants were in favor of CHTC after it was described to them, citing building trust, allowing for communication, and keeping partners “*on the same page*” as the main perceived benefits. One male participant said, *“I think one of the best, bigger benefits is stronger trust, like that’s a great benefit because y’all both know, y’all on the same page, and y’all both know what’s going on… that’s checked off. Now we can move on to the next thing*” (male). Several men reported recently seeking individual HIV counseling and testing and sharing the results with their partners. Women reported that CHTC would allow both members of the couple to be treated for a sexually transmitted infection (STI) at the same time: “*If it so happens that something is wrong and needs to be medicated, you can both make sure that you’re taking the medication correctly and follow up with going to make sure that the medication took care of the issue afterwards*” (female).

Participants reported that couples in more committed relationships – those married or engaged – were the most likely to utilize CHTC, whereas casual sex partners were the least likely: “*They don’t know how far their relationship is going to go, so what they want to go to the doctor for?*” (female) Extramarital relationships or very casual partners were described as less likely to use CHTC. Couples that were described as likely to get tested were generally in a committed relationship that was in a transition. Several types of transitions that were cited to favor couples testing were mentioned. One was recommitting after a break-up: “*That’s a good time to bring it up. We broke up for a month and we about to get this thing back together, it’s easier to pop that question [about getting tested together] at this time*” (male). Ceasing condom use in a committed relationship was another transition that was cited as being an appropriate time to utilize CHTC: *“Because if they went through that process of using condoms then at the same time they’re still being protective of each other, and testing is a good way to know if to move away from that protection”* (female). Another transition was planning for a deeper commitment or to have children: “*I would stay future planning would be a reason to, like if they plan on getting married, they plan on having children*” (female).

Discussing sexual agreements during CHTC was generally accepted as a beneficial practice:

“*Doing that together to take steps to, you know, do other things together that’s important to keep the relationship like glue I think*” (female).

“*Some of the benefit is that while we in the room together, she trusting me and I’m trusting her. This letting her know that I’m not cheating around on you, I’m not running around, if we getting tested together, we know what each other got, know*” (female).

Participants said that despite the potential for embarrassment or awkwardness, CHTC would be a good place to begin a discussion about agreements within the presence of a professional. One male participant said: “*If they both came together to be tested and then they sat down with the counselor and each one of them is asking the couxxnselors question, now they both will realize just how—if they pay attention—they’ll realize how the other one thinks, that may make it a little easier for them to communicate with one another*” (male).

### Barriers to CHTC

Although participants were supportive of CHTC, two barriers to the successful adoption of CHTC were mentioned. Underlying commitment to the relationship and an ability to communicate were described as prerequisite to CHTC. However, a suggestion of using CHTC outside the context of a transition or previously established explicit agreement that involved outside sex partners was cited as potentially eliciting suspicion of infidelity. One female participant said: “*It might be a little funny to all of a sudden, after 15 years, be like you want to go get counsel for HIV and AIDS*” (female). Confidentiality was reported as a major barrier to using CHTC, particularly if results were returned as sero-discordant. Participants noted that this would not be an issue for established couples, but would be a barrier to CHTC use among early stage couples or casual sex partners: *“See, I’m not just going in there talking about I’m gonna take a test with just anybody. If I go in there with somebody, it’s gonna be somebody that I know”* (female). Both men and women were concerned that sero-discordant results could also raise questions about fidelity:

“*Well automatically the person who is negative is going to assume that the person that they, that has it was the one cheating*” (female).

“*If you gonna go in there with somebody, make sure that that’s somebody loves you enough to respect you and if she loves you enough to respect you she’ll let you go before she try to hurt you*” (male).

### Suggestions for CHTC adaptation

Participants reported that rapport with the counselor was an important element of their comfort with the idea of CHTC, and suggested that there be opportunities to meet with the counselor and establish some level of relationship prior to undergoing CHTC: *“If they get to interact with that counselor initially, as opposed to coming back, because, you know, when you get someone telling you like how to, how to handle things in a relationship that you, I kind of feel like that should be someone that you got a little trust in”* (male). Additionally, one participant proposed that couples maintain the same counselor for future visits. Participants also spoke about the importance of having a counselor who had similar life experiences, did not take a particular partner’s side, and was non-judgmental. Appropriate advertising of CHTC was encouraged; participants noted that advertising of CHTC had to include images of heterosexual couples, and particular attention needed to be paid to encourage men to test with their female partners: “*Men need to go somewhere where they have a man mentor… Well they can see somebody has been through something, has been through the same stuff*” (female). Participants suggested advertising within family planning and STI clinics, but also at couples focused social venues. Participants also mentioned pregnancy as an opportune time for CHTC, and encouraged discussions of CHTC to be included in routine prenatal care visits.

## Discussion

The results presented here present preliminary qualitative data pointing to high levels of willingness among heterosexuals to use CHTC, with very few adaptations from the current CHTC protocol being used in the US. The most significant change in the CHTC protocol during the adaptation from the African heterosexual model to the US male-couple model was the addition of content requiring the couple to discuss their sexual agreement. This was added based on the known high prevalence of sexual agreements among male couples. Until now it was unknown whether asking heterosexual couples to discuss sexual agreements in a CHTC session would be acceptable and whether acceptability might vary by gender. . The results presented here – although from a small number of focus groups in one specific location – provide preliminary evidence that heterosexual couples would not only be willing to discuss sexual agreements during CHTC but also report the opportunity to have these discussions with a counselor present to be an important part of HIV prevention with their partner.

The focus group participants reported benefits to CHTC that were very similar to those reported in qualitative work with MSM in several US cities (Stephenson et al. 2010). Participants reported that CHTC may strengthen relationships by providing a forum for moderated discussion of HIV risk concerns, and would act to build and strengthen trust in relationships. Interestingly, participants reported that more committed couples – or couples who had been together for a longer period of time – would be more likely to use CHTC. This was also reported by MSM in the original qualitative work (Stephenson et al. 2010). The use of the term “couple” in CHTC is suggestive of a more committed, longer term dyad and in our experience of intervention roll-out we have found the CHTC term to be off-putting to individuals in newer relationships. CHTC is therefore commonly messaged as “Testing Together.” As the service develops popularity across the US, we are finding the couples of all relationship stages and lengths are using the service.

Participants constantly made a comparison between sexual agreements and “*understandings*,” with “*understandings*” being more pervasive than agreements. This suggests that in the delivery of the CHTC protocol, counselors may have to walk clients through this distinction; asking them first what their understanding of their sexual agreement is, if they also have a spoken agreement, and then facilitating them through the agreement building process based on their joint HIV test results and their relationship characteristics. Participants also noted the difficulty of bringing up CHTC in established relationships because it may lead to suspicion of infidelity; indeed, encouraging sexual risk reduction in established sexual partnerships has proven to be challenging for the field of HIV prevention because it implies a lack of trust and/or fidelity (Senn et al. 2014). Participants in the current study suggested that times of transition in a relationship, such as recommitting after a break up, discontinuing condom use, planning for children, or pregnancy would be opportune times to engage couples in CHTC.

The study has a number of key limitations. The results presented here are preliminary and are not generalizable to all heterosexuals, nor are they indicative of attitudes of all heterosexuals. The focus groups were conducted in one city only, and represent the local views of one small group of people. The number of focus groups is also small, and saturation across themes was not reached. Limited data on the characteristics of participants was collected, only age, race and relationship status. Information on relationship length, type of relationship and levels of commitment are necessary to fully understand how attitudes towards couples testing and sexual agreements may vary by relationship characteristics. However, in the absence of other data on the sexual agreements and attitudes towards CHTC among heterosexuals, they do provide useful preliminary data to inform the discussion around the adaptation and roll-out of CHTC for heterosexual couples in the US.

This small, preliminary qualitative study sheds new light on the potential for CHTC adoption among heterosexuals in the US. The results are similar to those seen in MSM: high levels of willingness to use CHTC, perceptions of the advantages of using CHTC, and willingness to discuss sexual agreements; all necessary conditions for the successful roll-out of CHTC. Further work is now needed with larger samples of high-risk heterosexuals to more completely understand the typologies of sexual agreements and the common language used for sexual agreements in heterosexual relationships. But these early data show great promise that CHTC can achieve the same levels of willingness, fit, and acceptability among heterosexual couples as currently experienced by male couples in the US.

## Abbreviations

CDC: Centers for disease control and prevention
CHTC: Couples HIV testing and counseling
EBI: Evidence-based intervention
FOCUS GROUP: Focus group discussion
IPV: Intimate partner violence
MSM: Men who have sex with men
